# Bathing

**Published:** 1872-04

**Authors:** 


### BATHING.

Many persons have lost their lives by getting chilled in the process of bathing; sometimes by going into the bath too soon after eating. No person should take any kind of bath sooner than three hours after a regular meal, and the room should show a heat of seventy-five degrees of Farenheit's thermometer, at about five feet above the floor, in the middle of the room, in order to avoid dangerous chills; persons of a feeble circulation should have the room still warmer; if there is an uncomfortable feeling of coldness to the body when it comes out of the water, the room is too cold.

